# Mitochondria-derived peptide MOTS-c restores mitochondrial respiration in type 2 diabetic heart

**DOI:** 10.3389/fphys.2025.1602271

**Published:** 2025-06-30

**Authors:** Toan Pham, Andrew Taberner, Anthony Hickey, June-Chiew Han

**Affiliations:** ^1^ Auckland Bioengineering Institute, The University of Auckland, Auckland, New Zealand; ^2^ Department of Engineering Science and Biomedical Engineering, The University of Auckland, Auckland, New Zealand; ^3^ School of Biological Sciences, The University of Auckland, Auckland, New Zealand

**Keywords:** MOTS-c, diabetic heart, mitochondrial respiration, ATP, reactive oxygen species

## Abstract

**Introduction:**

Type 2 diabetes (T2D) is a global epidemic, and heart failure is the primary cause of premature death among T2D patients. Mitochondrial dysfunction has been linked to decreased contractile performance in diabetic heart, partly due to a disturbance in the mitochondrial capacity to supply adequate metabolic energy to contractile proteins. MOTS-c, a newly discovered mitochondrial-derived peptide, has shown promise as a therapeutic for restoring energy homeostasis and muscle function in metabolic diseases. However, whether MOTS-c therapy improves T2D heart function by increasing mitochondrial bioenergetic function remains unknown.

**Methods:**

Here we studied the mitochondrial bioenergetic function of heart tissues isolated from a rat model mimicking type 2 diabetes induced by a high-fat diet and low-dose streptozotocin. Treated diabetic group received MOTS-c (15 mg/kg) daily injection for 3 weeks. We employed high-resolution respirometric and fluorometric techniques to simultaneously assess mitochondrial ATP production and hydrolysis capacity, reactive oxygen species (ROS) production, and oxygen flux in cardiac tissue homogenates.

**Results:**

We found that untreated T2D rats had hyperglycemia, poor glucose control, and left ventricular hypertrophy relative to controls. T2D mitochondria showed decreased oxygen flux at the oxidative phosphorylation (OXP) while ROS production, ATP production and hydrolysis rates remained unchanged. Diabetic rats treated with MOTS-c showed decreased fasting glucose levels, improved glucose homeostasis, and decreased degree of cardiac hypertrophy. At the subcellular level, MOTS-c treated mitochondria showed increased OXPHOS respiration and ROS levels and decreased ATP hydrolysis rate during anoxic conditions.

**Discussion:**

These findings demonstrate beneficial effects of MOTS-c treatment on glucose homeostasis and suggest a useful therapeutic option for diabetic-related cardiomyopathy and mitochondrial dysfunction.

## Introduction

Diabetes is rapidly becoming one of the most common chronic diseases. Over 90% of diabetic patients are diagnosed with Type 2 diabetes (T2D). Cardiovascular complications are the leading cause of morbidity and mortality in diabetic patients, with heart failure accounting for approximately 80% of all deaths (Boudina and Abel, 2007). Diabetic cardiomyopathy is defined by left ventricular hypertrophy, altered substrate metabolism (Amaral and Okonko, 2015; Stanley et al., 1997; Sharma et al., 2004) and, eventually, decreased contractile function (Marciniak et al., 2014; Montaigne et al., 2014). Mitochondrial dysfunction may precede contractile dysfunction and could be a key contributor to the decreased myocardial contractility seen in T2D patients (Montaigne et al., 2014; Anderson et al., 2009), as well as in rodent models of diabetes (Marciniak et al., 2014; Li et al., 2022; Tang et al., 2023). Understanding the underlying processes of diabetes-induced heart dysfunction is critical for establishing effective therapeutic options, yet it also remains an ongoing challenge for healthcare and biomedical research.

The heart is a high-energy-demanding organ and requires a continuous supply of adenosine triphosphate (ATP) to support constant mechanical work. Mitochondria primarily supply ATP via oxidative phosphorylation and occupy approximately 35% of the total volume of mammalian cardiac myocytes (Barth et al., 1992). Mitochondrial dysfunction often coincides with heart failure (Zhou and Tian, 2018). Emerging evidence has reported mitochondrial structural abnormalities (Tang et al., 2023; Boudina et al., 2007) and decreased bioenergetic function (Marciniak et al., 2014; Boudina et al., 2007; Croston et al., 2014; Pham et al., 2014) in diabetic hearts. As diabetes progresses, the heart decreases glucose utilisation, eventually relying almost exclusively on fatty acid (FA) oxidation to sustain ATP production (Amaral and Okonko, 2015; Stanley et al., 1997; Sharma et al., 2004). FA oxidation is less efficient than glucose oxidation, as it consumes more oxygen per unit of ATP generated (Lopaschuk et al., 2010). It also appears that increased reliance on FA oxidation in diabetic heart mitochondria associates with increased reactive oxygen species (ROS) production (Pham et al., 2014; Ye et al., 2004) and increased mitochondrial uncoupling protein expression (Boudina et al., 2007; Cole et al., 2011), which results in a decrease in the proton motive force and decreased ATP synthesis capacities (Pham et al., 2014; Ye et al., 2004).

MOTS-c is a newly discovered mitochondria-derived peptide encoded by the open reading frame in mitochondrial 12S rRNA. This small 16-amino-acid protein is highly conserved among species (Lee et al., 2015). Emerging data suggest that MOTS-c regulates cellular metabolism through AMPK-dependent pathways, enhancing glucose utilisation and stress responses (Lee et al., 2015; Kim et al., 2017; Merry et al., 2020a). MOTS-c is found in blood and other mitochondria-containing tissues. Blood MOTS-c levels were reported to be lower in T2D individuals (Ramanjaneya et al., 2019), gestational diabetes (Yin et al., 2022), coronary endothelial dysfunction (Qin et al., 2018), obese children and adolescents (Du et al., 2018), and streptozotocin-induced diabetic mice (Xu et al., 2024). Exogenous MOTS-c therapy has emerged as a promising therapeutic strategy for enhancing muscle mitochondrial function in metabolic disorders. In cell culture studies, MOTS-c administration has been demonstrated to increase mitochondrial ATP content in doxorubicin-induced senescent human fibroblasts (Kim et al., 2018) and HEK293 cells (Lee et al., 2015), as well as protecting against inflammation and oxidative stress in H9C2 cells (Shen et al., 2022). In animal studies, MOTS-c therapy has been shown to have a variety of positive benefits, including the prevention of age-related metabolic dysfunction (Li et al., 2019), ovariectomy-induced metabolic disturbances (Lu et al., 2019), insulin resistance in high-fat diet-induced obesity (Lee et al., 2015), and pressure overload-induced cardiac hypertrophy (Zhong et al., 2022). Recent research has also shown that MOTS-c treatment improved heart function in diabetic rats by enhancing glucose metabolism (Li et al., 2022; Yin et al., 2022) and upregulating antioxidant defences (Tang et al., 2023). However, the direct effect of MOTS-c therapy in restoring mitochondrial bioenergetic dysfunction in diabetic hearts is unknown.

We hypothesise that MOTS-c therapy may alleviate diabetic myocardial injury by improving mitochondrial respiration, increasing ATP production, and lowering oxidative stress levels. This study examined how MOTS-c treatment changed cardiac mitochondrial function in a well-established T2D rat model. This model has been shown to closely resemble the heart metabolic abnormalities seen in diabetic patients (Mansor et al., 2013; Guo et al., 2018). High-resolution respirometry and fluorometry were employed to investigate the mitochondrial bioenergetic function in cardiac tissue homogenates.

## Methods

### Animals

All experiments were conducted following protocols approved by the University of Auckland Animal Ethics Committee (AEC22653). Male Wistar rats (6–7 weeks old, 150–200 g) were randomly assigned to three groups: control (n = 12), untreated diabetic (n = 7), and treated diabetic (n = 11). Diabetes was induced using a combination of dietary and pharmacological interventions. Rats were fed with a high-fat diet (HFD, 43% digestible energy from lipids, SF04-001, Specialty Feeds, Australia) for a total duration of 15 weeks and received a low-dose intraperitoneal injection of streptozotocin (STZ, 25 mg/kg, in citrate buffer pH 4) at week 8 post-HFD. Control rats received standard chow and an injection of citrate buffer. At week 12, the treated diabetic group received daily intraperitoneal injections of MOTS-c (15 mg/kg/day) for 3 weeks, while the untreated diabetic and control groups received saline injections. All rats had water and food access in a 12-h light/dark cycle. Body weight and blood glucose were monitored weekly. At week 15, each rat was subjected to a glucose tolerance test following a 6-h fast before receiving an intraperitoneal injection of glucose bolus (2 g/kg, in sterile saline) and blood samples were obtained from the tail puncture. Blood glucose was measured using an Accu-Chek blood glucose meter at various time points (before the glucose tolerance test, then 15, 30, 60, 90, and 120 min after glucose administration).

### Drug

Mitochondria-derived peptide MOTS-c (MRWQEMGYIFYPRKLR) was synthesised by GenScript Biotech Ltd, Singapore and kept in powder form at −80 °C. MOTS-c was freshly dissolved in sterile saline before injection and used within 30 min.

### Tissue preparation

On the experiment day, rats were deeply anesthetised with isoflurane and injected with heparin (1,000 IU/kg). Following cervical dislocation, the heart was dissected, submerged in cold Tyrode solution, and then Langendorff-perfused with oxygenated Tyrode solution at room temperature. The Tyrode solution contained (in mmol/L): 130 NaCl, 6 KCl, 1 MgCl_2_, 0.3 CaCl_2_, 0.5 NaH_2_PO_4_, 10 HEPES, 10 glucose, and 20 2,3-butanedione monoxime (pH 7.4, adjusted with Tris). Ventricular free wall thickness, heart mass, and tibial length were measured.

Mitochondrial respiration was measured in fresh left ventricular samples. Fresh heart tissues (approximately 30 mg) were transferred to 750 µL cold MiRO5 buffer (without MgCl_2_), which contained (in mmol/L) 110 sucrose, 60 K-lactobionate, 20 HEPES, 20 taurine, 10 KH_2_PO_4_, 0.5 EGTA, 1 g/L BSA fatty acid free, and pH 7.1 adjusted at 30 °C. A small amount of MgCl_2_ (1 mmol/L) was used during the experiment to avoid signal interference with Magnesium Green fluorescence-based ATP measurement. Cardiac tissue samples were minced with small scissors, homogenised for 10 s at medium speed (Omni International, Hennesaw, GA) and transferred to oxygraph chambers for mitochondrial experiments. The remaining homogenate samples were stored at −80 °C for later enzymatic analysis.

### Mitochondrial experiments

Mitochondrial respiration was measured using a high-resolution fluo-respirometer (O2k, Oroboros Instruments, Innsbruck, Austria). This device has two separate 2-mL chambers. Each chamber has a polarographic oxygen sensor and a sealed stopper for substrate-inhibitor titration. Two detachable fluorimeters (Oroboros Instruments) were inserted in front of each chamber window to simultaneously measure the fluorescence of specific fluorophores (as described below) and O_2_ consumption rate. O_2_ tension was calibrated at an air saturation level (at 101 kPa) before each experiment. All measurements were conducted at 37 °C while the stirring speed was set to 750 rpm. All substrates, uncouplers, and inhibitors were added manually using Hamilton syringes.

#### Protocol 1: ATP production and hydrolysis

Magnesium Green (MgG, cell impermeant, M3733 Thermo Fisher Scientific) fluorescence (excitation: 470 nm, emission: 520 nm) was used to measure ATP-ADP exchange (Pham et al., 2014; Power et al., 2014) in a modified MiRO5 buffer (without MgCl_2_). MgG (5 μmol/L) and MgCl_2_ (1 mmol/L) were added before adding homogenate samples (2 mg) and waiting for signal stability. Complex I (CI) substrates, including malate (2 mmol/L), glutamate (10 mmol/L), and pyruvate (5 mmol/L) were used to induce leak respiration, which reflects O_2_ consumption compensating ion leaks without ATP synthesis, despite the presence of a small amount of ADP (∼0.03 μmol/L) in homogenate samples might contribute to negligible oxidative phosphorylation (OXPHOS) -linked respiration. ADP (2.5 mmol/L) was added to measure NADH-linked OXPHOS respiration in the presence of CI substrates, followed by succinate (10 mmol/L) to activate both CI and CII OXPHOS. Tissues were exposed to anoxia for 20 min to measure the maximal rate of ATP hydrolysis, followed by opening the stoppers to equilibrate with air to restore O_2_ concentration. Oligomycin (2 μmol/L) was added to inhibit ATP synthase, to return the MgG signal level to baseline, where this fluorescence level corresponded to no ATP synthesis activity by the mitochondria, allowing estimation of ATP-independent MgG signal. Antimycin A (1 μmol/L) was used to inhibit complex III (CIII) and allowed the measurement of non-mitochondrial O_2_ consumption.

#### Protocol 2: ROS production

Amplex UltraRed (AUR, 25 μmol/L), together with horseradish peroxidase (HRP, 10 U) and superoxide dismutase (SOD, 10 U), were used to assess mitochondrial H_2_O_2_ production (Pham et al., 2020; Hedges et al., 2020). Exogenous SOD converts superoxide radicals into H_2_O_2_, which then reacts with HRP and AUR to create resorufin (excitation: 525 nm, emission: 550 nm). H_2_O_2_ signal was calibrated with three times the known amount of H_2_O_2_ (122 μmol/L). MgCl_2_ (1 mmol/L) and homogenate samples (2 mg) were added to each chamber. CI substrates (malate, glutamate, pyruvate) and CII substrate (succinate) were added to initiate leak respiration linked to CI and CII. ADP (2.5 mmol/L) stimulated OXPHOS respiration from both CI and CII, while multiple titrations of carbonyl cyanide m-chlorophenyl hydrazone (CCCP, 1 μmol/L) assessed uncoupled respiration, demonstrating the maximal capacity of electron transfer system. Antimycin A (1 μmol/L) inhibited complex III to determine residual OCR. Complex IV (CIV) respiration was measured using ascorbate (2 mmol/L) and N,N,N′,N′-tetramethyl-p-phenylenediamine dihydrochloride (TMPD, 0.5 mmol/L), followed by inhibition with azide (100 mmol/L).

#### Protocol 3: fatty acid oxidation

MgCl_2_ (1 mmol/L) and homogenate samples (2 mg) were added to each chamber. To assess fatty acid metabolism, malate (0.1 mmol/L) and palmitoyl-L-carnitine (40 μmol/L) were used to initiate leak respiration. ADP (2.5 mmol/L) stimulated fatty acid-mediated OXPHOS respiration, followed by succinate (10 mmol/L) for CII-mediated OXPHOS. Antimycin A (1 μmol/L) was added to determine non-mitochondrial OCR.

### Citrate synthase activity

A protease inhibitor cocktail (25 μL, cOmplete Mini EDTA-free, Roche, Mannheim, Germany) was added to frozen homogenate samples. The samples were then thawed, vortexed, and centrifuged at 5,000 g for 10 min at 4 °C. Supernatants were collected and diluted 25-fold in phosphate-buffered saline to assess citrate synthase (CS) activity. Absorbance was measured at 412 nm every 30 s for a duration of 10 min with a microplate reader at room temperature in Tris-HCl buffer (50 mmol/L, pH 8) containing oxaloacetate (0.5 mmol/L), acetyl-CoA (0.1 mmol/L), and 5,5-dithiobis-(2-nitrobenzoic acid) (0.2 mmol/L). The CS activity was determined from the absorbance slope using an extinction coefficient value of 13,600 L/mol/cm and normalised for protein content. Total protein concentration was measured with a BCA protein kit (Thermo Fisher Scientific, Waltham, MA, USA).

### Data analyses

Respirometric and fluorometric data were recorded and analysed offline using DatLab 7.1 (Oroboros Instruments). O_2_ flux data were corrected for residual O_2_ consumption following the addition of antimycin, except for CIV respiration, where O_2_ flux data derived from the autooxidation effect were subtracted for O_2_ flux after CIV inhibition with sodium azide addition. ATP flux signals were calibrated using separate assays without tissue samples, as previously described (Pham et al., 2014) and corrected for the background signal of ATP synthase inhibition by oligomycin. H_2_O_2_ measurements were corrected for background H_2_O_2_ levels, which were measured before adding homogenate samples. Data were normalised to tissue wet mass.

### Statistical analyses

Statistical analyses were performed with GraphPad Prism 10.0. All data are presented as mean ± SEM unless otherwise stated. One-way analysis of variance test was used to examine group differences in all data, except that two-way analysis of variance was used to analyse blood glucose data in the glucose tolerance test. If a significant difference (p < 0.05) was detected, Fisher’s least significant difference post-hoc test was used to identify pairwise differences. All graphs were created using Prism software.

## Results

### Morphological characteristics of the rats

**FIGURE 1:**
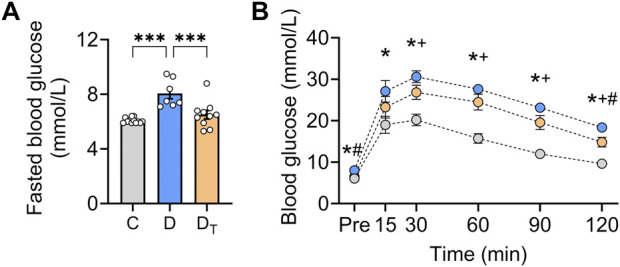
Glucose homeostasis in diabetic rats after MOTS-c treatment. **(A)** Fasted blood glucose levels. **(B)** Blood glucose levels were measured before and after a glucose bolus in control, untreated diabetic and treated diabetic rats. C, Control group (labelled grey), D, untreated diabetic group (labelled blue), D_T_: MOTS-c treated diabetic group (labelled orange). The colour of each of the three groups matches **(A)** Data are presented as means ± SEM. *p < 0.05 Control vs. untreated diabetic; ^+^ *p* < 0.05 control vs. treated diabetic; ^#^ *p* < 0.05 untreated diabetic vs. treated diabetic rats.

Untreated diabetic rats had notably higher fasting glucose levels than control and MOTS-c-treated diabetic rats, with no difference between the latter two groups (Figure 1A). We excluded rats with type 1 diabetic signs, including hyperglycaemia (>30 mmol/L), polyuria, and substantial weight loss. Untreated diabetic rats demonstrated decreased glucose clearance capability compared to controls, whereas MOTS-c-treated rats showed improved glucose handling, although blood glucose levels remained higher than the control group (Figure 1B).

Compared to the controls, the untreated diabetic rats exhibited increases in body mass, tibial length, heart mass and left-ventricular thickness, mimicking T2D symptoms (Table 1). Three weeks of MOTS-c treatment alleviated these increases. No statistical differences in any parameters were detected between the control group and the treated diabetic group.

**TABLE 1:** Morphological characteristics of the rats at the time of sacrifice.

Parameter	Control *(n = 12)*	Untreated diabetic *(n = 7)*	Treated diabetic *(n = 11)*
Body mass (g)	498.2 ± 11.7	538.7 ± 20.5*	511.7 ± 10.0
Heart mass (g)	1.56 ± 0.04	1.78 ± 0.09*	1.57 ± 0.02^#^
Tibial length (mm)	43.8 ± 0.4	45.0 ± 0.4*	44.1 ± 0.3
Left ventricular thickness (mm)	3.31 ± 0.05	3.71 ± 0.06*	3.4 ± 0.05^#^
Right ventricular thickness (mm)	1.38 ± 0.03	1.51 ± 0.07	1.49 ± 0.05
Heart mass per body mass	0.0031 ± 0.0001	0.0033 ± 0.0001	0.0031 ± 0.0001
Heart mass per tibial length (g/mm)	0.036 ± 0.001	0.04 ± 0.002*	0.036 ± 0.001^#^
LV thickness per tibial length	0.076 ± 0.001	0.082 ± 0.001*	0.077 ± 0.001^#^

Values are means ± SEM.

*p < 0.05 Control vs. untreated diabetic.

^#^ *p* < 0.05 untreated diabetic vs. treated diabetic.

### Mitochondrial respiration

**FIGURE 2:**
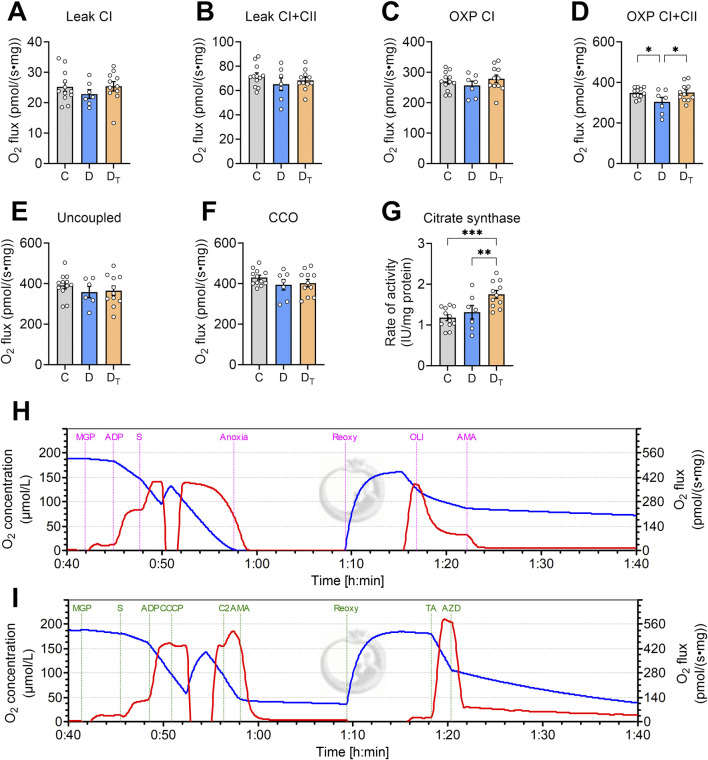
Mitochondrial O_2_ flux measured from left ventricular homogenates. O_2_ flux is normalised to tissue wet weight **(A–F)**. **(G)** Citrate synthase activity as a measure of mitochondrial content, IU unit is in µmol/min. Representative traces of O_2_ concentration (blue) and O_2_ flux per tissue mass (red) from Protocol #1 **(H)** and Protocol #2 **(I)**. CI: complex I, CII, complex II, OXP: oxidative phosphorylation, CCO: cytochrome C oxidase, CCCP: carbonyl cyanide m-chlorophenyl hydrazone, TA: N, N, N′, N′-tetramethyl-p-phenylenediamine dihydrochloride and ascorbate, C, Control group, D, untreated diabetic group, D_T_, MOTS-c treated diabetic group. All data are presented as mean ± SEM.

Untreated diabetic heart mitochondria showed no significant differences in O_2_ consumption rate or O_2_ flux from carbohydrate-supported substrates (addition of malate, glutamate, and pyruvate) at CI and CII leak, uncoupled respiration, or CIV respiration states compared to other groups (Figure 2). However, diabetic mitochondria had a lower mitochondrial O_2_ flux in CI and CII OXPHOS state compared to the control and MOTS-c-treated diabetic groups, with no difference between the latter two groups (Figure 2D). The activity of CS normalised to protein content was significantly higher in the MOTS-c-treated diabetic group than in the control and untreated diabetic groups, indicating that the MOTS-c-treated group had increased mitochondrial content (Figure 2G).

**FIGURE 3:**
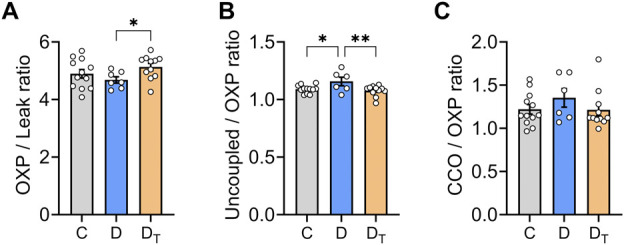
Mitochondrial respiratory flux control ratios. **(A)** The respiratory control ratio, a measure of mitochondrial coupling efficiency, was calculated using both Complex I and Complex II substrates with and without ADP. **(B)** Uncoupling control ratio was calculated as a ratio of O_2_ flux in uncoupled respiration state compared to oxidative phosphorylation (OXP) state. **(C)** Activity of cytochrome c-oxidase (CIV) relative to the coupled OXP state. C, Control group, D, untreated diabetic group, D_T_, MOTS-c treated diabetic group. Data are expressed as mean ± SEM.

Figure 3 presents data on the respiratory control ratio, which measures mitochondrial coupling efficiency, defined as the ratio of O_2_ flux in the OXPHOS state and O_2_ flux in the leak state. MOTS-c treated diabetic mitochondria exhibited a significantly higher respiratory control ratio than the untreated group (Figure 3A). Untreated diabetic mitochondria had a higher uncoupling control ratio than the control and MOTS-c treated groups (Figure 3B). There were no significant differences in cytochrome c-oxidase or CIV activity relative to the coupled OXPHOS state among the groups (Figure 3C).

**FIGURE 4:**
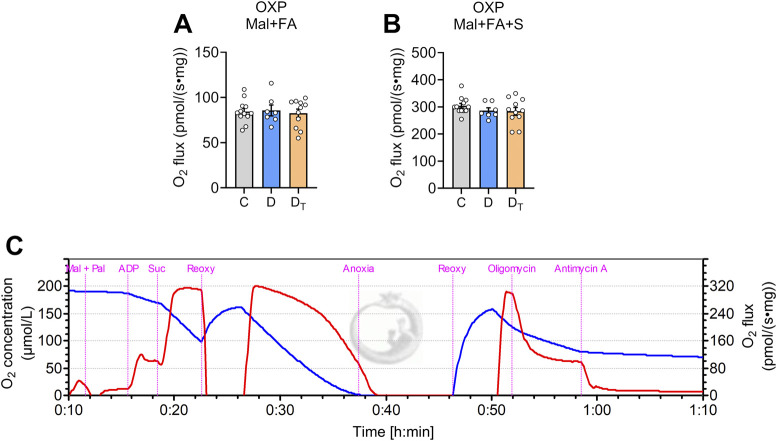
Respiration capacity of mitochondrial palmitoyl-carnitine oxidation. Mitochondrial O_2_ flux measured from left ventricular homogenates normalised to tissue wet weight **(A,B)** Representative traces of O_2_ concentration (blue) and O_2_ flux per tissue mass (red) from Protocol #3 **(C)** Mal, malate, Pal, palmitoyl-L-carnitine, Suc, succinate, Reoxy, reoxygenation, C, Control group, D, untreated diabetic group, D_T_, MOTS-c treated diabetic group. Data are expressed as mean ± SEM.

Figure 4 shows data of mitochondrial respiration supported by a fatty acid-based substrate (palmitoyl-L-carnitine) and a low concentration of malate. When normalised to tissue wet mass, mitochondrial fatty acid oxidation-mediated respiration was similar among groups.

### Steady-state ATP synthesis and hydrolysis measurements

**FIGURE 5:**
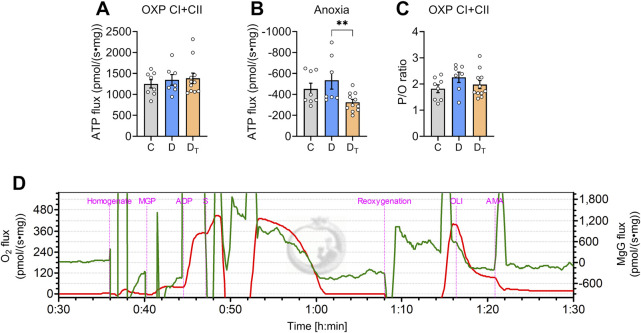
ATP production and hydrolysis capacity in left ventricular homogenates. Rates of ATP production per tissue mass in normoxic **(A)** and anoxic [**(B)** negative values] conditions. **(C)** Steady-state P/O ratio was calculated using the rate of ATP production relative to the oxygen flux in oxidative phosphorylation (OXP) respiration state. **(D)** Representative traces of O_2_ flux per tissue mass (red) and MgG signal flux (Green) from Protocol #1. C, Control group, D, untreated diabetic group, D_T_, MOTS-c treated diabetic group. Data are expressed as mean ± SEM.

When normalised to tissue wet mass, ATP production rate in the OXPHOS state did not differ among groups (Figure 5). Similarly, no significant differences were observed in the P/O ratio, a measure of mitochondrial energy efficiency, when expressed relative to steady-state O_2_ flux (Figure 5C). During anoxia, ATP synthase operates in reverse, consuming ATP and resulting in negative ATP flux, and no difference was observed between control and untreated diabetic groups (Figure 5B). However, MOTS-c treated diabetic mitochondria exhibited significantly lower ATP hydrolysis rates than untreated diabetic groups.

### H_2_O_2_ production measurements

**FIGURE 6:**
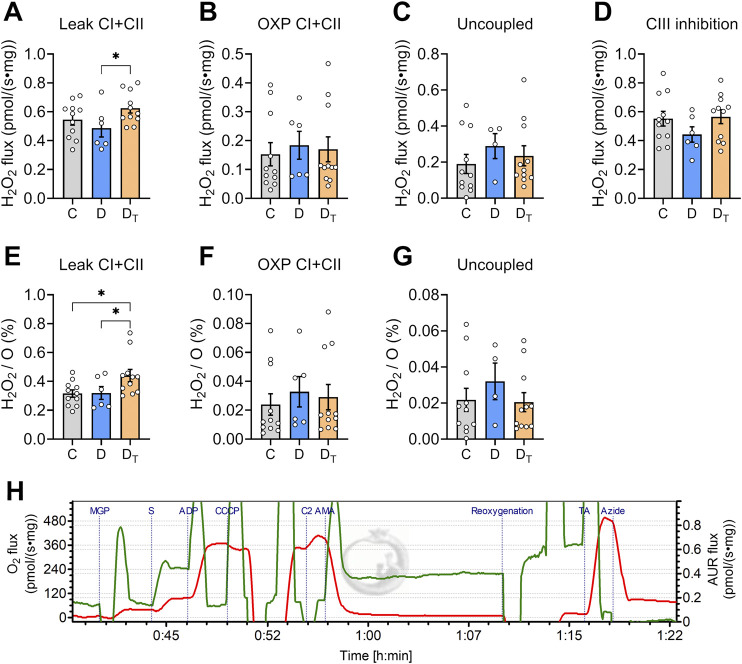
H_2_O_2_ production rates in left ventricular homogenates. **(A–D)** H_2_O_2_ production rate per tissue mass at various respiration states; **(E–G)** H_2_O_2_ production relative to oxygen flux (H_2_O_2_/O). **(H)** Representative traces of O_2_ flux per tissue mass (red) and AUR signal flux (Green) from Protocol #2. CI, complex I, CII, complex II, OXP, oxidative phosphorylation, C, Control group, D, untreated diabetic group, D_T_, MOTS-c treated diabetic group. Data are expressed as mean ± SEM.

H_2_O_2_ production at various respiratory states did not differ between control and untreated diabetic groups (Figure 6). MOTS-c-treated diabetic mitochondria exhibited higher H_2_O_2_ production per tissue mass at the CI and CII leak state than untreated diabetic mitochondria (Figure 6A). At the same CI and CII leak state, H_2_O_2_ production expressed as a percentage of single oxygen flux (H_2_O_2_/O) was significantly higher in treated diabetic mitochondria than in control and untreated diabetic groups (Figure 6E).

## Discussion

This study examined the effects of MOTS-c on mitochondrial bioenergetic function in Type 2 diabetic hearts. Our findings reveal that untreated diabetic rats gained more weight, had poorer glucose regulation, developed left-ventricular hypertrophy and had lower mitochondrial respiration at the OXPHOS respiratory state compared to the control group. MOTS-c treatment significantly lowered weight gain, improved glucose handling, and reversed cardiac hypertrophy. MOTS-c improved carbohydrate-supported mitochondrial respiration and increased citrate synthase activity. However, MOTS-c treated diabetic mitochondria decreased the capacity of ATP hydrolysis during anoxia while having no effect on FA-supported mitochondrial respiration, mitochondrial ATP production and ROS production in CI-CII OXP state.

### Animal morphological phenotypes

In this study, we employed a T2D-like rat model induced by a combination of a high-fat diet and low-dose STZ injection, which has been shown to resemble cardiac metabolic abnormalities in diabetic individuals (Mansor et al., 2013; Guo et al., 2018). In this study, we confirmed that rats on this regimen increased body weight and had poor glucose homeostasis. Rats with substantial weight loss and hyperglycaemia, which resembled Type 1 diabetes, were excluded from this study. Our findings demonstrate that MOTS-c treatment effectively delayed weight gain in diabetic rats, which is consistent with recent research in HFD-induced obese mice (Lee et al., 2015) and T1D mice (Xu et al., 2024). These investigations also found that MOTS-c treatment did not affect food intake, indicating that MOTS-c may directly increase whole-body metabolic rate.

MOTS-c treatment has been demonstrated to lower blood glucose levels in T1D mice (Xu et al., 2024), T1D rats (Li et al., 2022), or gestational diabetic mice (Yin et al., 2022). Our findings extend these reports as MOTS-c treatment decreased fasted blood glucose levels and improved glucose handling in T2D rats. We also observed an 8% decrease in left ventricular wall thickness in the treated diabetic group, highlighting the potential beneficial effects of MOTS-c on restoring cardiac dysfunction, as consistently reported in previous studies on T1D model (Li et al., 2022; Wu N. et al., 2023). Previous studies reported that MOTS-c treatment induces activation of AMPK signalling pathway, which improves insulin sensitivity and upregulates GLUT4 expression in skeletal muscle, enabling efficient glucose uptake and metabolism (Lee et al., 2015; Yin et al., 2022).

### Carbohydrate- and FA-supported mitochondrial respiration

In untreated diabetic rats, mass-specific oxygen flux from carbohydrate-supported substrates (including malate, glutamate and pyruvate) was 13% lower in CI + CII OXP respiratory state than in the control. This observation is consistent with decreased mitochondrial respiration found in our previous study in T1D rat model (Pham et al., 2014) and others in T2D mouse model (Boudina et al., 2007). Our data in this present work show that MOTS-c treatment restores mitochondrial respiration per tissue mass, aligning with previous findings of increased mitochondrial respiration in senescent cells (Kim et al., 2018). The increased respiration following MOTS-c treatment may be attributed to increased mitochondrial content rather than intrinsic functional gain. It is possible that MOTS-c treatment increased CS activity per each mitochondrion without increasing total mitochondrial volume, which warrants further investigation to confirm this assumption. Nevertheless, our findings are consistent with previous findings in T1D rats (Tang et al., 2023; Wang et al., 2022) reported increases in both CS activity and mitochondrial number, and preserved mitochondrial structure in the treated diabetic group. This is likely attributed to increased activation of the AMPK pathway to induce increased mitochondrial biogenesis biomarkers (Xu et al., 2024) in response to MOTS-c treatment.

Additionally, untreated diabetic mitochondria had a greater uncoupled respiratory ratio, suggesting impaired phosphorylation efficiency despite preserved electron transfer system (ETS) capacity. This apparent uncoupling was also restored by MOTS-c treatment. CIV oxygen flux, which represents the terminal step of the ETS, remained unchanged across groups. Given that CIV flux typically exceeds ETS flux in most mitochondria (Pham et al., 2020; Po et al., 2016), our findings indicate that neither T2D nor MOTS-c treatment altered maximal oxygen flux through CIV activity (Figure 2J).

Compared to glucose oxidation, fatty acid oxidation is less efficient as it generates less ATP per oxygen unit consumed (Barclay and Loiselle, 2020). Plasma levels of free fatty acids and triglycerides have been reported to be higher in diabetic patients (Anderson et al., 2009) and rats (Tang et al., 2023). Interestingly, despite increased fatty acid transport in diabetic hearts (Bonen et al., 2009; Chabowski et al., 2008; Ljubkovic et al., 2019), maximal fatty acid-supported mitochondrial respiration was found to be unchanged in left ventricular tissues from T2D patients (Ljubkovic et al., 2019), or decreased in atrial tissues from T2D patients (Anderson et al., 2009; Croston et al., 2014) and left ventricular tissue in a T1D mouse model (Boudina et al., 2007), indicating a maladaptive metabolic response to abundant FA availability. Our data reveal no differences in mitochondrial respiration from fatty acid-supported substrates among groups, consistent with the report in T2D patient ventricular tissues (Ljubkovic et al., 2019), suggesting that the effect of T2D on FA-mediated mitochondrial respiration could be tissue region-dependent. We further note that the homogenate samples used in this study may contain some levels of endogenous fatty acids, which could confound our findings. Future research would focus on changes in fatty acid transport proteins and substrate oxidation pathways, and the use of isolated mitochondria to better understand energy substrate metabolism in both ventricular and atrial tissues of diabetic hearts.

### ATP production

In this study, we assessed ATP production and mitochondrial respiration at the OXPHOS state to calculate the P/O ratio, the indicator of mitochondrial energy efficiency. Using various electron inputs from CI and CII substrates, the P/O ratio provides information about mitochondrial ATP synthesis and turnover capabilities. We found no differences in maximal ATP production rate or P/O ratio across groups, showing mitochondrial energy efficiency remained preserved both in diabetes and under treatment. This contradicts previous reports of lower mitochondrial ATP production in T1D rats (Pham et al., 2014), T2D mice (Boudina et al., 2007), and systemic hypertensive rats (Po et al., 2016). Interestingly, only T1D hearts (Pham et al., 2014) had a lower mitochondrial P/O ratio, highlighting disease-specific bioenergetic alterations. Previous research in senescent cell cultures (Lee et al., 2015; Kim et al., 2018) reported higher ATP content, most likely resulting from increased glucose uptake (Lee et al., 2015). However, our findings show no change in ATP production rate in MOTS-c treated diabetic mitochondria, suggesting that increased ATP content could arise from increased glycolysis pathway (Lee et al., 2015; Kim et al., 2018) instead of the oxidative phosphorylation process.

Real-time ATP measurements enabled us to determine ATP consumption during anoxia (Goo et al., 2013). Under anoxic circumstances, mitochondria halt electron transport and proton pumping while ATP synthase reverses to hydrolyse ATP to balance ionic homeostasis. Our data indicate that the mitochondrial ATP hydrolysis rate was significantly lower in MOTS-c treated diabetic group compared to untreated diabetic group. In other words, MOTS-c moderated lower ATP consumption during anoxia without compromising ATP production in normoxia. It has been demonstrated that diabetic hearts are more susceptible to ischemic insults (Paulson, 1997). Our findings suggest a protective role of MOTS-c in conserving ATP during ischemia while maintaining myocardial ATP levels during post-ischemic recovery.

### H_2_O_2_ production

We simultaneously quantified mitochondrial H_2_O_2_ output and oxygen consumption rates at different respiratory states. Under conditions of high succinate and limited ADP, which simulate ischemic conditions (Chouchani et al., 2014), H_2_O_2_ production increased rapidly due to superoxide production at CI via reverse electron transport. In this respiratory state, the MOTS-c treatment group generated a higher H_2_O_2_ production, and this was more pronounced when expressed as a percentage of O_2_ flux (Figure 6I), compared to controls. Interestingly, transient bursts in H_2_O_2_ production may be important signals for cellular adaptation against stress (Ristow and Zarse, 2010; Yun and Finkel, 2014). The initial action of MOTS-c treatment could increase antioxidant responses by increasing protein expressions of superoxide dismutase, catalase, and glutathione peroxidase 4, as reported in a previous study on T1D hearts (Tang et al., 2023). This response is mediated through Nrf2 activation as a key regulator of antioxidant gene transcription (da Costa et al., 2019; Chen and Maltagliati, 2018; Merry et al., 2020b). This upregulated antioxidant defence system may protect cardiomyocytes from additional damage arising from increased oxidative stress, a known primary driver of diabetic cardiomyopathy pathogenesis (Anderson et al., 2009; Boudina et al., 2007; Pham et al., 2014; Ye et al., 2004).

### MOTS-c doses and treatment duration

The biological activity and therapeutic effects of MOTS-c treatment are highly dose-dependent, resulting in significant variations in experimental results. Currently, no defined MOTS-c dosage ensures optimal treatment effects across different diseases. The literature describes various MOTS-c dosages, each generating diverse biological reactions. The reported doses include 0.5 mg/kg (Li et al., 2022; Yuan et al., 2021; Yang et al., 2024), 1 mg/kg (Xu et al., 2024), 5 mg/kg (Wu N. et al., 2023; Li et al., 2024; Jiang et al., 2023), 7.5 mg/kg (Zempo et al., 2021), 10 mg/kg (Yin et al., 2022), 15 mg/kg (Reynolds et al., 2021; Wu J. et al., 2023; Kumagai et al., 2024), or 50 mg/kg (Yin et al., 2020). Generally, lower doses (0.5–5 mg/kg) were usually taken over 8–12 weeks of treatment, whereas higher doses (10–15 mg/kg) were used in shorter periods, within 2–4 weeks. It is important to consider potential side effects associated with higher MOTS-c doses, including injection site responses and digestive disturbances. Our study, using the MOTS-c dose of 15 mg/kg daily over 3 weeks, was based on a previous study showing beneficial effects of MOTS-c in a similar timeframe and dose range (Reynolds et al., 2021). Future research is encouraged to explore the ideal MOTS-c dosage and treatment duration, all of which balance therapeutic efficacy and safety.

### Conclusion

T2D rats exhibited impaired glucose-handling ability, gained more weight compared to the control group, and developed cardiac hypertrophy. T2D heart mitochondria showed decreased mitochondrial respiration without mitochondrial ATP production or ROS production changes. MOTS-c treatment improved glucose handling and reversed cardiac hypertrophy in diabetic rats. The MOTS-c treatment further restored mitochondrial respiration by increasing the mitochondrial content and decreasing the ATP hydrolysis rate. Our findings suggest that MOTS-c may be a promising treatment for mitochondrial dysfunction in diabetic settings.

## Data Availability

The raw data supporting the conclusions of this article will be made available by the authors, without undue reservation.
